# Development and Validation of KASP Assays for the Genotyping of Racing Performance-Associated Single Nucleotide Polymorphisms in Pigeons

**DOI:** 10.3390/genes12091383

**Published:** 2021-09-03

**Authors:** Ching-Chi Chang, Benji Brayan I. Silva, Huai-Ying Huang, Ching-Yi Tsai, Ronilo Jose D. Flores, Lemmuel L. Tayo, Yu-Chang Tyan, Ming-An Tsai, Gail Everette M. Catulin, Kuo-Pin Chuang, Jenq-Lin Yang

**Affiliations:** 1 Graduate Institute of Animal Vaccine Technology, College of Veterinary Medicine, National Pingtung University of Science and Technology, Pingtung 912, Taiwan; m10824005@mail.npust.edu.tw (C.-C.C.); vetlaenwe@gmail.com (H.-Y.H.); j10785002@g4e.npust.edu.tw (C.-Y.T.); 2 International Degree Program in Animal Vaccine Technology, International College, National Pingtung University of Science and Technology, Pingtung 912, Taiwan; g10985004@mail.npust.edu.tw (B.B.I.S.); yctyan@kmu.edu.tw (Y.-C.T.); 3 Demin Veterinary Hospital, Kaohsiung 807, Taiwan; 4 Institute of Biological Sciences, College of Arts and Sciences, University of the Philippines Los Baños, Laguna 4031, Philippines; rdflores3@up.edu.ph; 5 Graduate School, University of the Philippines Los Baños, Laguna 4031, Philippines; 6 School of Chemical, Biological and Materials Engineering and Sciences, Mapúa University, Intramuros, Manila 1002, Philippines; lltayo@mapua.edu.ph (L.L.T.); gemcatulin@mymail.mapua.edu.ph (G.E.M.C.); 7 School of Medicine, College of Medicine, Kaohsiung Medical University, Kaohsiung 807, Taiwan; 8 Institute of Medical Science and Technology, National Sun Yat-Sen University, Kaohsiung 804, Taiwan; 9 Department of Medical Imaging and Radiological Sciences, Kaohsiung Medical University, Kaohsiung 807, Taiwan; 10 Department of Medical Research, Kaohsiung Medical University Hospital, Kaohsiung 807, Taiwan; 11 Research Center for Environmental Research, Kaohsiung Medical University, Kaohsiung 807, Taiwan; 12 Department of Veterinary Medicine, National Pingtung University of Science and Technology, Pingtung 912, Taiwan; matsai@mail.npust.edu.tw; 13 International Program in Ornamental Fish Technology and Aquatic Animal Health, International College, National Pingtung University of Science and Technology, Pingtung 912, Taiwan; 14 Research Center for Animal Biologics, National Pingtung University of Science and Technology, Pingtung 912, Taiwan; 15 School of Dentistry, Kaohsiung Medical University, Kaohsiung 807, Taiwan; 16 Institute for Translation Research in Biomedicine, Kaohsiung Chang Gung Memorial Hospital, Kaohsiung 807, Taiwan

**Keywords:** *DRD4* gene, genetic resources, KASP, *LDHA* gene, *MTCYB* gene, PCR-RFLP, polymorphism

## Abstract

Pigeon racing’s recent upturn in popularity can be attributed in part to the huge prize money involved in these competitions. As such, methods to select pigeons with desirable genetic characteristics for racing or for selective breeding have also been gaining more interest. Polymerase chain reaction—restriction fragment length polymorphism (PCR-RFLP) for genotyping-specific genes is one of the most commonly used molecular techniques, which can be costly, laborious and time consuming. The present study reports the development of an alternative genotyping method that employs Kompetitive Allele Specific Polymerase Chain Reaction (KASP) technology with specifically designed primers to detect previously reported racing performance-associated polymorphisms within the *LDHA*, *MTYCB*, and *DRD4* genes. To validate, KASP assays and PCR-RFLP assays results from 107 samples genotyped for each of the genes were compared and the results showed perfect (100%) agreement of both methods. The developed KASP assays present an alternative rapid, reliable, and cost-effective method to identify polymorphisms in pigeons.

## 1. Introduction

Domesticated, bred, and kept for centuries, pigeons (*Columba livia*) have literally been physically altered by human interventions that purposively select characteristics based on the following three general reasons: (1) utility, for meat and guano, and historically, as a messenger; (2) fancy, for their visual and physical appearance; and (3) sporting/flying, for recreation and competitions. Among these three reasons, sporting is the most common reason for breeding pigeons nowadays [1,2,3,4]. Pigeon racing is a popular and lucrative sport that attracts competitors around the world and involves millions of dollars in prices during competitions [2,3,5,6,7,8].

With races ranging from 60 to 310 miles, pigeons are bred and selected for their homing ability or spatial orientation, which would dictate the pigeons’ success to return to their home loft after being released from a specific distance. Additionally, higher overall capacity in terms of endurance and recovery, or speed, velocity, and stress resistance are preferred traits [2,3,6,8,9,10,11]. Besides health management programs, including the appropriate choice of feeds, medical supplements, and physical conditioning regimens to improve pigeons’ performance in competitions, the identification of genetic markers for valuable traits associated with racing performance has also been reported and explored towards expediting success in selective breeding [1,6,8,9,10,11,12,13,14,15,16,17,18,19]. The determination of single nucleotide polymorphisms (SNPs) that seem to cause phenotypic differences is one of the most commonly used approaches to identify potentially essential genetic markers [20,21,22,23]. Genotyping based on SNPs may be executed through several methods, among which polymerase chain reaction—restriction fragment length polymorphism (PCR-RFLP) is the most commonly performed. This method involves the amplification of a target gene that would then be subjected to different restriction enzymes to recognize and cut the DNA molecules at particular sites, thus generating a characteristic banding pattern [23,24,25].

Using PCR-RFLP, previously reported studies have identified SNPs in several genes as potential markers for the racing performance of pigeons. Of note among these reports are the significant differences of the frequencies of certain SNP alleles in the lactate dehydrogenase A (*LDH-A*), mitochondrial cytochrome B (*MTCYB*), and the dopamine receptor D4 (*DRD4*) genes when comparing homing and non-homing pigeon populations, thus suggesting potential associations of these SNPs with racing performance [1,9,19,26]. To date, PCR-RFLP has been used for individual selection of pigeons for racing and breeding; however, the use of this method is laborious and slow, thus only conveniently applicable for basic research purposes with a relatively smaller number of samples [25,27]. A low-cost, efficient, and scalable alternative method that can give accurate results is needed.

Among recent developments in SNP genotyping techniques, the use of Kompetitive Allele Specific PCR (KASP) is becoming more common. This technology utilizes two allele-specific forward primers, each of which has an additional unique tail sequence that corresponds with a universal FRET (fluorescence resonant energy transfer) cassette. The PCR reactions generate the tail sequence complement of the relevant allele specific forward primer. The fluorescence-labeled oligonucleotides from the reporter cassette bind to the tail sequence complement, thus releasing the FRET cassette from the quencher that enables the emission of the fluorescent signal. The sample genotype is determined by identifying which of the only two possible signals was generated, or if both signals are generated. A single fluorescent signal means homozygosity for its corresponding SNP allele, while a mixed signal denotes heterozygosity [28,29,30,31]. 

While KASP assays have found applications in species identification, and genetic markers/polymorphisms detection for both plants and animals [20,29,30,31,32,33,34,35,36,37,38,39,40,41], no study has yet employed this technology for pigeon SNP genotyping. Aimed at providing a new tool that may help improve pigeon breeding and management, this study developed KASP assays for SNP genotyping within three race performance-associated genes and validated their accuracies against PCR-RFLP.

## 2. Materials and Methods

### 2.1. Samples and Sample Collection

Feather samples were collected from a pigeon farm in Pingtung, Taiwan. All of the pigeons from which loose feather samples were collected were subject to the same breeding and health management practice of the farm. Freshly molted down or contour feather samples from 107 individuals were collected and stored in sealed bags that were maintained at −20 °C until DNA extraction. DNA was extracted using the Viogene Geno Plus™ DNA extraction miniprep system (Viogene-BioTek Corporation, Taipei, Taiwan), and the extracts were stored at −20 °C until further use.

### 2.2. PCR-RFLP Genotyping

A total of four polymorphisms within three different genes—*LDHA*, *MTCYB*, and *DRD4*—were investigated in this study. The presence of one polymorphism within each of the *LDHA* and *MTCYB* genes, and two polymorphisms within the *DRD4* gene, from hereon referred to as *DRD4* (1) and *DRD4* (2), were assayed for detection. All PCR cocktails used were prepared as follows: 4.0 µL of genomic DNA at 5–50 ng/μL, 0.2 µL of each primer at 100 ng/μL, 5.0 µL of 2X PCR buffer (LGC, UK), and 0.6 µL water to a total volume of 10.0 µL. Amplifications of the target genes were then carried out using a DNA thermal cycler (Blue-Ray Biotech Corp., Taipei, Taiwan) under the following conditions: initial denaturation at 96 °C for 10 s, followed by multiple cycles (32 cycles for *LDHA* and *DRD4* (1), and 35 cycles for the others) of denaturation at 96 °C for 15 s, annealing at variable temperature (57 °C for *LDHA*, 65 °C for *MTCYB*, 66 °C for *DRD4*) for 15 s and extension at 72 °C for 15s, and a 5 min final extension at 72 °C. The primers used are shown in Table 1.

After PCR, the amplified portions of the target genes were subjected to enzymatic digestion under the following conditions: for the *LDHA*, the PCR amplicon was digested by incubation with HaeIII (New England Biolabs, Ipswich, MA, USA) at 37 °C for 160 min; for *MTCYB*, the PCR amplicon was digested by incubation with MvaI (New England Biolabs, Ipswich, MA, USA) enzyme at 60 °C for 240 min; for *DRD4* (1) and *DRD4* (2), the PCR amplicons were digested by incubation with HpyCH4III (New England Biolabs, Ipswich, MA, USA) (Bst4CI and HpyCH4III are isoschizomers) for 6 h and MnlI (New England Biolabs, Ipswich, MA, USA) for 3 h. The digested fragments were separated and viewed by gel electrophoresis using 2% agarose gels (Amresco, Solon, OH, USA) in 1× Tris-borate-EDTA (TBE) at 100 volts for 30 min.

### 2.3. KASP Assay

The KASP assay for single SNP includes two allele specific forward primers and one common reverse primer which were designed and validated by LGC Genomics (Herts, UK). The assay mix preparation and PCR amplifications were performed according to the user’s guide and manual (LGC Genomics, Hoddesdon, Herts, UK). The concentration of DNA samples was detected using NanoDrop2000 spectrophotometer (Thermo Fisher Scientific, Waltham, MA, USA) and was diluted for genotyping, as required. The assays were performed in a 96-well plate containing 10 µL reaction volume, which consist of 5 µL 2 × KASP Master Mix, 0.14 µL KASP primer mix, and 4.86 µL genomic DNA at 5–50 ng/µL. The plates were then sealed with adhesive film (Micro-Amp Optical Adhesive Film, Thermo Fisher Scientific, Waltham, MA, USA) and centrifuged at 5000g for 30s. KASP genotyping was carried out on a StepOnePlusTM real-time PCR system (Thermo Fisher Scientific, Waltham, MA, USA) using the following cycling conditions: pre-read stage at 30 °C for 60 s, next stage at 94 °C for 15 min, followed by 10 cycles at 94 °C for 20 s, 61 °C (decreasing by 0.6 °C per cycle) for 60 s, and then 35 cycles at 94 °C for 20 s, 55 °C for 60 s, and finally, post-read stage at 30 °C for 60 s. Primers used are shown in Table 2.

## 3. Results

The study included a total of 107 individuals sampled for genotyping using two different techniques—PCR-RFLP and KASP. Four SNPs in three different genes were detected using previously reported PCR-RFLP protocols, while four separate KASP assays were developed and validated. The results of the two assays were compared for similarity. PCR-RFLP is regarded as the standard protocol against which the accuracy of the KASP assays were validated.

**Figure 1:**
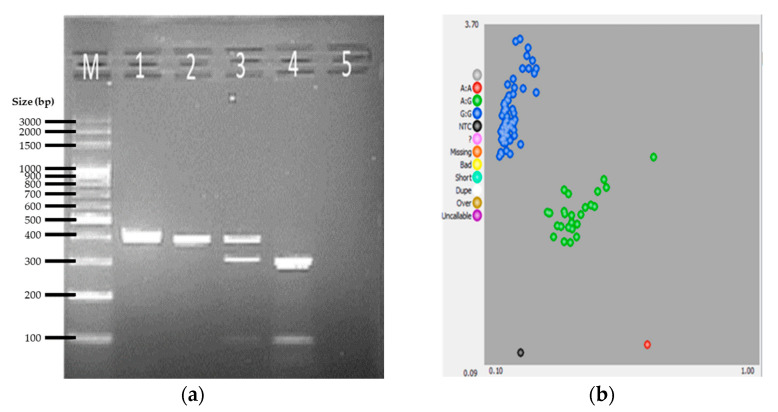
Representative results for *LDHA* gene SNP genotyping by PCR-RFLP and KASP assays. (**a**) Agarose gel electrophoretic profile of selected samples showing the banding patterns for the undigested PCR amplicon (lane 1), the *LDHA^AA^* allele (lane 2), *LDHA^AG^* allele (lane 3), and *LDHA^GG^* allele. (**b**) Cluster plot for the KASP genotyping assay: *LDHA^AA^* allele (red cluster), *LDHA^AG^* allele (green cluster), and *LDHA^GG^* allele (blue cluster).

First, a previously identified SNP within the LDHA gene (g.2582481G>A), of which the homozygous AA genotype was previously reported to be significantly linked to racing performance of pigeons, was investigated in this study [2,6,9]. The PCR amplification yielded a 423 bp product. Subsequent digestion of this amplicon with HaeIII generated three banding patterns corresponding to three genotypes: LDHA^AA^—395 bp, 28 bp; LDHA^AG^—395 bp, 311 bp, 84 bp, 28 bp; and LDHA^GG^—311 bp, 84 bp, 28 bp. Among the 107 samples tested, 80 were identified as genotype LDHA^GG^, while 26 and 1 were genotyped as LDHA^AG^, and LDHA^AA^, respectively (Table 3). Samples of the PCR-RFLP band patterns for the detection of the target SNP in the LDHA gene are shown in Figure 1a. Testing for the same SNP in the LDHA gene of the same 107 individual samples using KASP assay resulted in three distinct clusters representing the three genotypes, as shown in the plot (Figure 1b). Perfect similarity between the PCR-RFLP and KASP assay for LDHA was observed (Table 3). 

**Figure 2:**
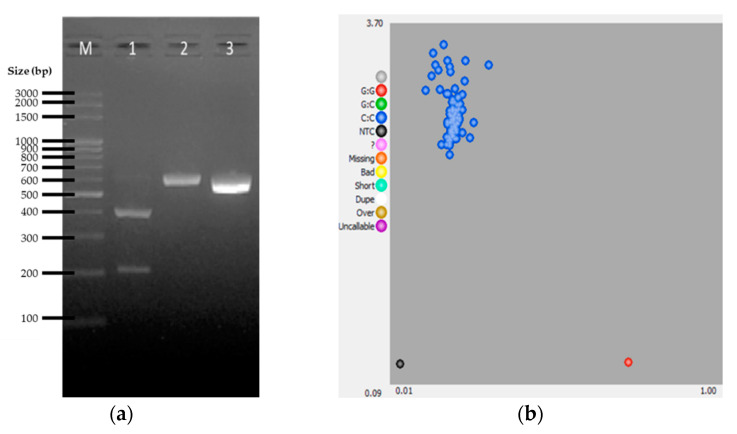
Representative results for *MTCYB* gene SNP genotyping by PCR-RFLP and KASP assays. (**a**) Agarose gel electrophoretic profile of selected samples showing the banding patterns for the *MTCYB^G^* allele (lane 1), and *MTCYB^C^* allele (lane 2), and the undigested PCR amplicon (lane 3). (**b**) Cluster plot for the KASP genotyping assay: *MTCYB^G^* allele (red cluster), and *MTCYB^C^* allele (blue cluster).

Following [26], the MTCYB/MvaI polymorphism was also investigated. Observation frequencies of the alleles of this silent mutation were found to be considerably deviating when homing and non-homing pigeon populations were compared. Notably, the MTCYB^C^ allele was only observed among non-homing breeds [26]. The PCR produced an amplicon of 608 bp. Digestion of the PCR products of the 107 samples using MvaI generated two banding patterns. A profile showing 405bp and 203bp indicated that the sample has the MTCYB^G^ allele, while the profile showing no digestion (608bp) indicates that the sample has the MTCYB^C^ allele (Figure 2a). The cluster plot (Figure 2b) of the allele calling by KASP assay showed only one group corresponding to the detection of MTCYB^C^ allele. Perfect similarity between the PCR-RFLP and KASP assay for MTCYB was also observed (Table 3).

Among the four reported SNPs in the *DRD4* gene [19], KASP assays for the intronic mutation g. 129954C > T (ss. 1751581452) and the missense mutation g. 129456C > T (p. Leu175Phe; ss. 1751581454), which are referred to in this paper as *DRD4* (1) and *DRD4* (2), respectively, were developed. These two SNPs were selected based on their reported association to racing performance as measured by pigeon ace points. Comparing individuals identified to be homozygous (CC) for the *DRD4* (2) SNP, heterozygotes (CT) were observed to have acquired significantly more ace points in short distance races. Additionally, the combination of the genotypes of both the *DRD4* (1) and *DRD4* (2) loci were also found to influence racing performance. Notably, as CTCT combined genotype was linked to highest average ace point, so was the CCCC genotype to lowest average ace point.

**Figure 3:**
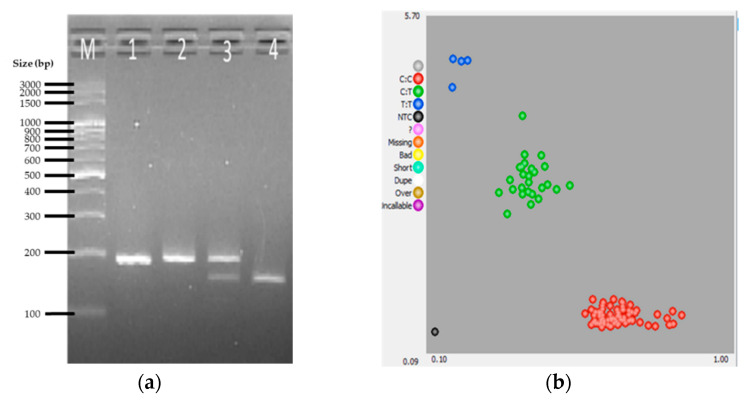
Representative results for *DRD4* (1) gene SNP genotyping by PCR-RFLP and KASP assays. (**a**) Agarose gel electrophoretic profile of selected samples showing the banding patterns for the undigested PCR amplicon (lane 1), the TT allele (lane 2), TC allele (lane 3), and CC allele (lane 4) (**b**) Cluster plot for the KASP genotyping assay: TT allele (blue cluster), TC allele (green cluster), and CC allele (red cluster).

For *DRD4* (1), the PCR-RFLP assay of the 107 samples resulted in the detection of three genotypes: TT, characterized by a band of size 191bp (undigested by HpyCH4III); TC, with 191bp, 153bp, and 38bp fragments; and CC, with 153bp and 38bp fragments. Figure 3a shows the five representative PCR-RFLP banding pattern product among the 107 samples. Additionally, there were 77, 26, and 4 samples genotyped as CC, CT, and TT, respectively, 100% of which agreed with the KASP assay results (Figure 3b, Table 3). 

**Figure 4:**
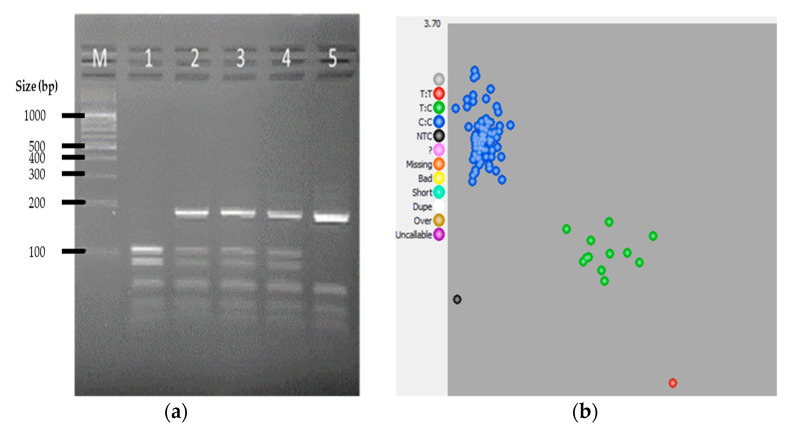
Representative results for *DRD4* (2) gene SNP genotyping by PCR-RFLP and KASP assays. (**a**) Agarose gel electrophoretic profile of selected samples showing the banding patterns for the CC allele (lane 1), TC allele (lanes 2, 3, and 4), and TT allele (lane 5). (**b**) Cluster plot for the KASP genotyping assay: TT allele (red cluster), TC allele (green cluster), and CC allele (blue cluster).

Lastly, among the same 107 samples, three *DRD4* (2) genotypes were also observed using both PCR-RFLP and KASP assays. As shown in Figure 4a, digestion of the 294 bp PCR products using MnlI generated fragments with sizes 182 bp, 54 bp, 35 bp, 23 bp indicating genotype TT; 182 bp, 101 bp, 81 bp, 54 bp, 35 bp, 23 bp for genotype TC; and 101 bp, 81 bp, 54 bp, 35 bp, and 23 bp for genotype CC. Both assays resulted in the identification of 94 samples as genotype CC, while 12 samples were genotype TC, and one sample was genotype TT. The summative comparison of PCR-RFLP and KASP assay results for genotyping selected race performance-associated SNPs in pigeons is shown in Table 3.

## 4. Discussion

With the growing popularity of pigeon racing as a lucrative sport, molecular approaches to the identification of genetic markers, such as SNPs, important to racing and homing phenotypes have been explored in the past [1,2,3,5,6,7,8,12,13,17,26,42]. The results of this study demonstrated the application of KASP as a method to detect SNPs previously identified to be associated with the racing traits of pigeons. With 100% similarity to the results of PCR-RFLP, it is shown that KASP assays can be used as an accurate alternative method to the slow and laborious traditional techniques to perform SNP genotyping, particularly PCR-RFLP, which may have been useful when dealing with studies with smaller sample sizes [25,27]. 

For the four PCR-RFLP assays tested in this report, the price difference as compared to each KASP assay counterpart is heavily dictated by the cost of the restriction enzyme used for digestion. For instance, HpyCH4III is about four times more expensive per unit than HaeIII. Our comparison of the material costs (excluding the initial investment for the PCR machines) necessary to perform PCR-RFLP and KASP assays showed that a PCR-RFLP assay can be about 10–100% more expensive compared to a single KASP assay testing for the same polymorphism.

Additionally, the total execution time of both protocols also differs significantly. While KASP assays typically only need 2.5 h to perform from DNA extraction to interpretation of the results, PCR-RFLP assays spend around 7 h to do the same genotyping analysis. Thus, if the execution time is to be included in the costing analysis since this would correspond to hours of work of technicians to perform the assays, KASP would, all in all, cost about three times less than PCR-RFLP.

The limitations and constraints of the more traditional genotyping techniques therefore necessitate the development and validation of new methods that would make the implementation of such analyses more accessible to more potential beneficiaries, such as pigeon racers, fanciers, and breeders, and genetic researchers alike. In a recent report [42], the use of quantitative polymerase chain reaction with high-resolution melting (qPCR-HRM) post-amplification offered a new approach to the resolve the limitations of conventional PCR to the study of another kind of polymorphism, also present and relevant to racing pigeons, called microsatellite polymorphism. Reporting about (TTTAT)_3–5_ microsatellite polymorphism in intron 6 of the LDHA gene, [42] described the usability of qPCR-HRM to resolve the “stutter-bands or shadow bands” and heteroduplexes problems of conventional PCR among heterozygous samples. Similar to the current study, this highlights the availability of space and opportunity in the field of genetic research for the development, validation, and advancement of alternative approaches to solve the limitations of older techniques [42].

The validated KASP genotyping assays are expected to facilitate an improved breeding and selection process for racing pigeons. As in [42], the currently reported method may also potentially contribute to the advancement of our understanding of polymorphisms and their relationship with observable characteristics. As more potential genetic markers are being identified to be related to desirable traits among racing pigeons [2,10,12,17,42], and considering the advantages of KASP assays compared to PCR-RFLP in terms of cost, speed, and scalability as discussed above, this study may also serve as a basis for the further development of other KASP assays to detect other single nucleotide polymorphisms in pigeons, and even in other avian species. Lastly, to the best of our knowledge, this is the first report of the use of KASP assays in any avian species.

## Figures and Tables

**Table 1:** Primers used for PCR-RFLP.

Gene	Sequence	Restriction Enzyme	Reference
*LDHA*	F 5′-TGAAGGGGTACACATCATGG-3′ R 5′-CCTTCTGGATTCCCCAGAGT-3′	HaeIII	[9]
*MTCYB*	F 5′-TTTGGGTCCCTACTAGGCATT-3′ R 5′-GAGGACAAGGAGGATGGTGA-3′	MvaI	[26]
*DRD4* (1)	F 5′-TTTGGGATCGCTCGCTTACC-3′ R 5′-ATGACAGGGGATGCTACAGC-3′	HpyCH4III	[19]
*DRD4* (2)	F 5′-GGGCCAACAGGAAGCTCTAT-3′ R 5′-GCAGGACAACACAGCGTCTC-3′	MnlI	[19]

**Table 2:** Primer sequences for KASP assays.

Gene	KASP Assay	Position	Sequences (5′–3′)	Gene ID
*LDHA*	Allele-G Forward Primer	52–76	ATCTCTACAGTTGTTAAGGTGAGCG	MW072294.1
	Allele-A Forward Primer	51–76	AATCTCTACAGTTGTTAAGGTGAGCA	
	Common Reverse Primer	122–94	CCAAGGTTTTTAGGTCTCAGTAAGACAAA	
*MTCYB*	Allele-C Forward Primer	14314–14336	ACTTCTCCCTAAAAGACATCCTC	NC013978.1
	Allele-G Forward Primer	14312–14336	CTACTTCTCCCTAAAAGACATCCTG	
	Common Reverse Primer	14371–14347	AGGGTCATTAGGGGGAGGAGTATTA	
*DRD4* (1)	Allele-T Reverse Primer	45–26	GAGCCAGGCCCAGGGTACTA	MT982613.1
	Allele-C Reverse Primer	44–26	AGCCAGGCCCAGGGTACTG	
	Common Forward Primer	2–25	CGCTTACCTTACGAGCGGTGACAA	
*DRD4* (2)	Allele-C Forward Primer	524–504	CGACTGTCTCCTATCCCCACC	MT982613.1
	Allele-T Forward Primer	524–504	CGACTGTCTCCTATCCCCACT	
	Common Reverse Primer	575–554	GGCCGTTGATCTTGGCCCGTTT	

**Table 3:** Summary of results for the genotype obtained using PCR-RFLP and KASP.

Gene	Genotype	PCR-RFLP	KASP	Percent Similarity
*LDHA*	AA	1	1	100%
	AG	26	26	100%
	GG	80	80	100%
*MTCYB*	G	1	1	100%
	C	106	106	100%
*DRD4* (1)	CC	77	77	100%
	CT	26	26	100%
	TT	4	4	100%
*DRD4* (2)	TT	1	1	100%
	TC	12	12	100%
	CC	94	94	100%

## Data Availability

The data presented in this study are contained within the article.
